# Health System Performance Assessment: how equitable is the Belgian health system?

**DOI:** 10.1093/eurpub/ckac129.244

**Published:** 2022-10-25

**Authors:** M Van de Voorde, N Bouckaert

**Affiliations:** Belgian Healthcare Knowledge Center, KCE, Brussels, Belgium

## Abstract

Access to and delivery of effective, high quality and affordable healthcare are fundamental objectives that have shaped health policy and the universal health insurance system in Belgium. A health system should be evaluated against these objectives. Monitoring equity within a health system - an equitable distribution of healthcare use and of payments for healthcare - is a core component of a health system performance assessment. We use the horizontal equity principle to evaluate equity in healthcare use in Belgium. Unfair inequality in healthcare use is measured by the fairness gap, comparing actual use and expected use corrected for needs. The empirical analysis was conducted using a linked dataset which contains data from the European Union Statistics on Income and Living Conditions (EU-SILC) and administrative data. Two sets of results are reported for a selected set of indicators of use: the deviation between the average fairness gap in the population and the average fairness gap in a subgroup of interest and an assessment of systematic socioeconomic inequity in the fairness gap using the absolute concentration index. We show that important socioeconomic inequities in healthcare use exist. Inequities differ by type of care, e.g., the use of hospital care and medications is more equitable than the use of GP, outpatient specialist and dental care. When accounting for healthcare needs, we find that use among high-income groups and individuals with a high educational attainment is higher compared to financially vulnerable groups (individuals at risk of poverty or with severe material deprivation, unemployed, singles). Also, individuals who are entitled to an increased reimbursement of healthcare costs, show a lower use of outpatient specialist care than expected based on their care needs. On the other hand, increased reimbursement is effective in improving accessibility to GP care, while for other financially vulnerable individuals we find a lower use of GP care.

